# Clinical features of pulmonary embolism in patients with lung cancer: A meta-analysis

**DOI:** 10.1371/journal.pone.0223230

**Published:** 2019-09-30

**Authors:** Xin Hua, Shu-Hua Han, Shu-Zhen Wei, Ying Wu, Jun Sha, Xiao-Li Zhu

**Affiliations:** 1 Department of Respiratory, Zhongda Hospital, Southeast University, Nanjing, Jiangsu, China; 2 Medical School of Southeast University, Nanjing, Jiangsu, China; National Cancer Center, JAPAN

## Abstract

**Background:**

Pulmonary embolism (PE) is correlated with increased mortality among patients with lung cancer (LC). The characteristics of patients with LC presenting with PE have not been fully established, and our meta-analysis aims to comprehensively investigate the clinical characteristics associated with PE in patients with LC to help physicians identify PE earlier in these patients.

**Methods:**

Multiple databases were searched, including PubMed, EMBASE, Cochrane Library, China National Knowledge Infrastructure and Wanfang. Odds ratios (ORs) and weighted mean differences (WMDs) with 95% confidence intervals (95% CIs) were used as effect measures for dichotomous and continuous variables, respectively. Moreover, Egger’s test, Begg’s test and a sensitivity analysis were performed to assess the publication bias and reliability of the articles.

**Results:**

In total, 16 studies were included in our meta-analysis. The results indicated that history of chronic obstructive pulmonary disease (OR = 2.59, 95% CI: 1.09, 6.15; P = 0.03), adenocarcinoma (OR = 2.28, 95% CI: 1.88, 2.77; P < 0.01), advanced tumour stage (TNM III-IV vs. I-II, OR = 2.38, 95% CI: 1.99, 2.86; P < 0.01), history of central venous catheter (OR = 1.95, 95% CI: 1.36, 2.78; P < 0.01), history of chemotherapy (OR = 2.32, 95% CI: 1.80, 2.99, P < 0.01), high levels of D-dimer (WMD = 4.31, 95% CI: 2.53, 6.10; P < 0.01) and carcinoembryonic antigen (WMD = 10.30, 95% CI: 9.95, 10.64; P < 0.01) and a low level of partial pressure of oxygen (WMD = -25.97, 95% CI: -31.31, -20.62; P < 0.01) were clinical features of LC patients with PE compared to those without PE.

**Conclusions:**

These results reveal that LC patients with PE have specific clinical features, including but not limited to several cancer- and treatment-related factors, that may help their early identification.

## Introduction

In 1868, Trousseau first identified a close connection between venous thromboembolism (VTE) and cancer [1]. Many recent studies have demonstrated that VTE is associated with worse outcomes in cancer patients and that VTE is the second leading cause of death after cancer among cancer patients [2–4]. VTE includes deep vein thrombosis (DVT) and pulmonary embolism (PE); the former often leads to post-thrombotic syndrome and the latter often results in more dangerous complications, such as pulmonary infarction and chronic thromboembolic pulmonary hypertension [5]. According to a previous study, lung cancer (LC) is the malignancy most commonly associated with PE [6]. In addition, multiple clinical studies have confirmed that the occurrence of PE is associated with an increased death rate among LC patients [7–10], accounting for 10% of deaths [11]. However, among LC patients with PE who receive early anticoagulation therapy, the mortality rate is decreased four-fold compared to that in patients who do not receive early therapy [12].

Therefore, early identification, diagnosis and treatment of PE could reduce mortality among LC patients. In fact, misdiagnosis or missed diagnosis of PE often occurs in a clinical setting because the clinical symptoms of PE are usually not readily recognizable [13]. According to a retrospective study, fatigue and shortness of breath are the most common symptoms among cancer patients with unsuspected PE, which is often not considered as a possible explanation [14]. Thus, investigating the clinical features associated with PE among LC patients is important.

Previous systematic reviews have described the association between LC and PE and summarized the relevant clinical features of PE in LC patients. For example, Malgor et al. [15] described that among LC patients with adenocarcinoma, chemotherapy appears to be associated with more frequent PE. In 2018, Li et al. [16] not only described the incidence, pathophysiological considerations, treatment and prognostic significance of PE among LC patients but also summarized the risk factors associated with PE and showed that adenocarcinoma, more advanced TNM stage, anaemia, obesity, history of chronic obstructive pulmonary disease (COPD), DVT, hospitalization in the 12 months before diagnosis of LC, surgery, chemotherapy, targeted drugs, central venous catheter (CVC), and haemoglobin (Hb), white blood cell (WBC), D-dimer (DD), partial pressure of oxygen (PaO_2_), and carcinoembryonic antigen (CEA) levels were all associated with PE in LC patients. Although the above studies used a comprehensive set of indicators, no consensus exists on the results, because they were based on univariate analyses and limited sample sizes.

As an effective statistical tool, meta-analysis can integrate the results of various independent studies and overcome the limitations of individual research, which often exhibit large heterogeneities and biases. Therefore, we conducted a meta-analysis of the published literature related to this topic to investigate the association between clinical characteristics and PE in LC patients and to clarify which associations are supported by sufficient epidemiological evidence to help physicians identify PE earlier in LC patients.

## Materials and methods

### Search strategy

This study was performed according to the recommendations of the Preferred Reporting Items for Systematic Reviews and Meta-Analyses (PRISMA) statement. Multiple databases, including PubMed, EMBASE, Cochrane Library, China National Knowledge Infrastructure (CNKI) and Wanfang, were systematically searched for related studies without a time restriction (from inception to February 2019). The search terms included “pulmonary embolism” or “pulmonary thromboembolism” and “lung cancer” or “lung carcinoma” separately and in combination. Moreover, we screened the references of pertinent studies to identify potentially related articles.

The following inclusion criteria were used: (1) studies with a prospective design, cohort design or case-control design; (2) studies comparing clinical features, including COPD history, pathological type, TNM stage, CVC history, chemotherapy history, WBC, and Hb, DD, platelet (PLT), PaO_2_, and CEA levels, and survival rate in LC patients with and without PE; (3) studies involving clear and standardized diagnostic criteria for LC and PE, including histologically or cytologically confirmed LC, or imaging findings, including echocardiography, computed tomography (CT), magnetic resonance imaging (MRI) or ventilation/perfusion (V/Q) scan, confirming PE; (4) studies published in English or Chinese with full-text articles that could be retrieved; (5) studies in which the sample size per group was greater than 30; and (6) studies reporting at least one outcome measure of interest or outcome measures that could be calculated from published data. In addition, we excluded meta-analyses, reviews, letters to the editor, case reports, reports based on expert experience and systematic reviews without a quantitative synthesis of the data. If more than one article referred to the same population, only the study that included the largest number of LC cases or the most recent publication was included to avoid duplication of the study populations. Two reviewers (HX and HSH) first independently screened the studies for possible inclusion based on the title and abstract and excluded irrelevant studies according to the above inclusion criteria. The full-text articles were further reviewed to evaluate potentially relevant studies.

### Data extraction and assessment of the study quality

For statistical analysis, the following data were retrieved from the studies: (1) basic aspects of the included studies, including the first author’s name, year of publication, characteristics of the study population (number, age, sex and TNM stage) and study design; (2) study-specific risk estimates: risk ratios, odds ratios (ORs) and mean differences along with their 95% confidence intervals (95% CIs) for clinicopathological information and the occurrence of PE, including COPD history, pathological type, TNM stage, CVC history, chemotherapy history, WBC, and Hb, DD, PLT, PaO_2_ and CEA levels; and (3) patient survival rate. Information that could not be extracted was described as “not mentioned (NM)”.

The quality of the included studies was assessed with the Newcastle-Ottawa Scale (NOS) [17], and a total score of at least 6 was considered high quality. Two reviewers (WY and SJ) independently evaluated the quality of the eligible conventional studies, and any disagreements were resolved by consensus or discussion with a third author (ZXL).

### Statistical analysis

We used Review Manager 5.3 and Stata 12.0 software to analyse the data in our meta-analysis. A related indicator was included in the meta-analysis if it was reported in at least 2 studies. The pooled ORs and weighted mean differences (WMDs) were calculated to evaluate the association between the occurrence of PE and the clinicopathological features of LC patients. For dichotomous variables, the ORs from multivariate models, with confounding factors adjusted in each study, were used. For continuous variables, WMDs were used to measure the effects. All statistical values are reported with the 95% CIs, and the two-sided P-value threshold for statistical significance was set at 0.05. The Chi-square test and I^2^ statistic were used to evaluate heterogeneity among the studies. In addition, P < 0.05 based on the Chi-square test or I^2^ greater than 50% suggested significant heterogeneity among the studies.

If the hypothesis of homogeneity was not rejected, a fixed-effects model was used; otherwise, a random-effects model was used to estimate ORs, WMDs and 95% CIs [18]. To explore the effects of individual studies on the overall results, we also performed a sensitivity analysis by excluding each study in turn. Finally, potential publication bias was assessed by Egger’s and Begg’s tests.

## Results

### Baseline study characteristics and quality assessment

**Fig 1:**
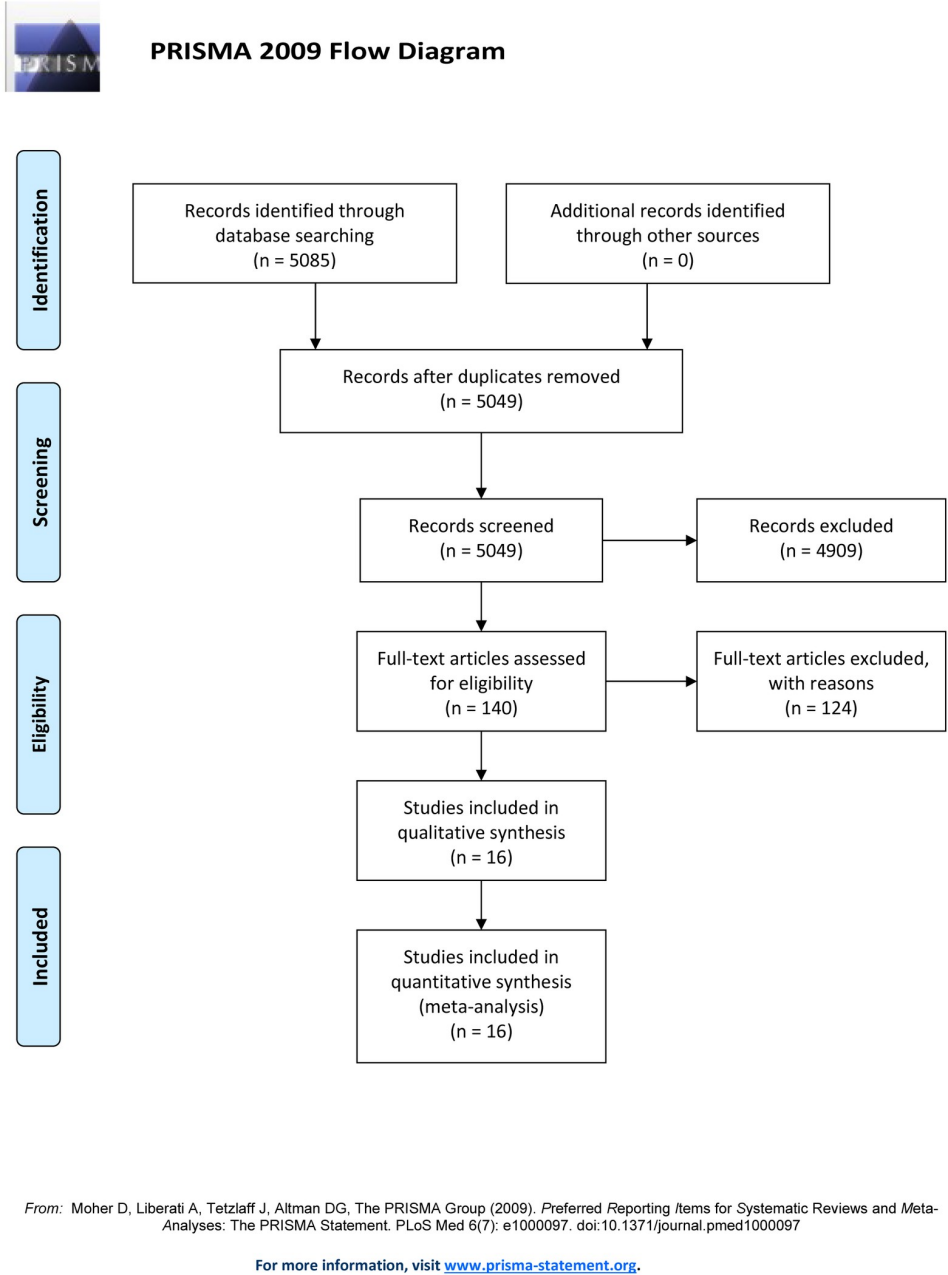
PRISMA flow diagram for this meta-analysis.

As shown in Fig 1, we identified 5085 studies in our systematic literature search, and 36 were excluded because they were repetitive. After reviewing the titles and abstracts, we retrieved 140 potential studies for review of the full text. Next, 124 studies were excluded because they lacked an outcome of interest and effective data or because they included fewer than 30 patients. Ultimately, 16 studies met our selection criteria for the final analysis. The characteristics and demographic data of all studies included are presented in Table 1 [12,13,19–32]. In total, 15305 patients were included, and the largest study involved 8015 patients. The retrieved studies were published between 2010 and 2018.

**Table 1:** Basic characteristics of the retrieved studies.

Study	Year	Number of patients		Age of patients		Sex of patients (m/N)		TNM stage	Study design	NOS
		PE + LC	LC	PE + LC	LC	PE + LC	LC			
Tie et al. [19]	2018	87	87	62.1±7.7	62.3±7.59	47/87	44/87	I-IV	Case control	7
Tang et al. [20]	2017	32	64	42~73	35~69	18/32	41/64	I-IV	Case control	5
Na et al. [21]	2017	35	80	57.9±8.1	56.0±9.8	21/35	44/80	I-IV	Case control	5
Tian et al. [22]	2017	32	32	55.3±7.5	54.1±8.9	22/32	24/32	I-IV	Case control	6
Zhao et al. [23]	2017	72	72	64.1±5.3	64.3±4.3	40/72	42/72	I-IV	Case control	6
Ai et al. [13]	2017	54	60	64.6±4.1	65.9±4.2	31/54	36/60	II-IV	Case control	5
Luo et al. [24]	2017	39	43	62.7±5.3	65.8±5.7	22/39	25/43	II-IV	Case control	6
Ma et al. [25]	2017	30	60	67.4±10.1	65.0±11.5	20/30	40/60	I-IV	Case control	7
Xiong et al. [26]	2017	1016	4064	65.5±18.6	63.9±17.9	428/1016	1666/4064	I-IV	Cohort	6
Zhu et al. [27]	2016	46	46	54.3±8.9	54.7±8.7	28/46	30/46	I-IV	Case control	5
Zhang et al. [28]	2015	57	57	63.6±9.8	62.8±0.2	34/57	36/57	I-IV	Case control	5
Shi et al.[29]	2015	35	105	62.5±10.1	60.6±10.5	20/35	60/105	I-IV	Case control	7
Xiong et al. [30]	2014	53	43	43.71±10.67	44.67±11.24	28/53	23/43	NM	Case control	5
Zhang et al. [31]	2014	47	626	NM	NM	37/47	449/626	I-IV	Cohort	7
Wang et al. [32]	2011	54	162	38~86	37~86	35/54	105/162	I-IV	Case control	6
Sun et al. [12]	2010	180	7835	NM	NM	115/180	5668/7835	I-IV	Cohort	7

**Abbreviations:** PE + LC: pulmonary embolism with lung cancer patients; LC: sample lung cancer patients; m/N: male/total number of patients; NOS, Newcastle-Ottawa Scale; NM, not mentioned.

For the quality assessment, the NOS was used to evaluate all 16 studies; 10 were evaluated as high quality and 6 as low quality (Table 1).

### Meta-analysis of the clinical characteristics of patients

**Fig 2:**
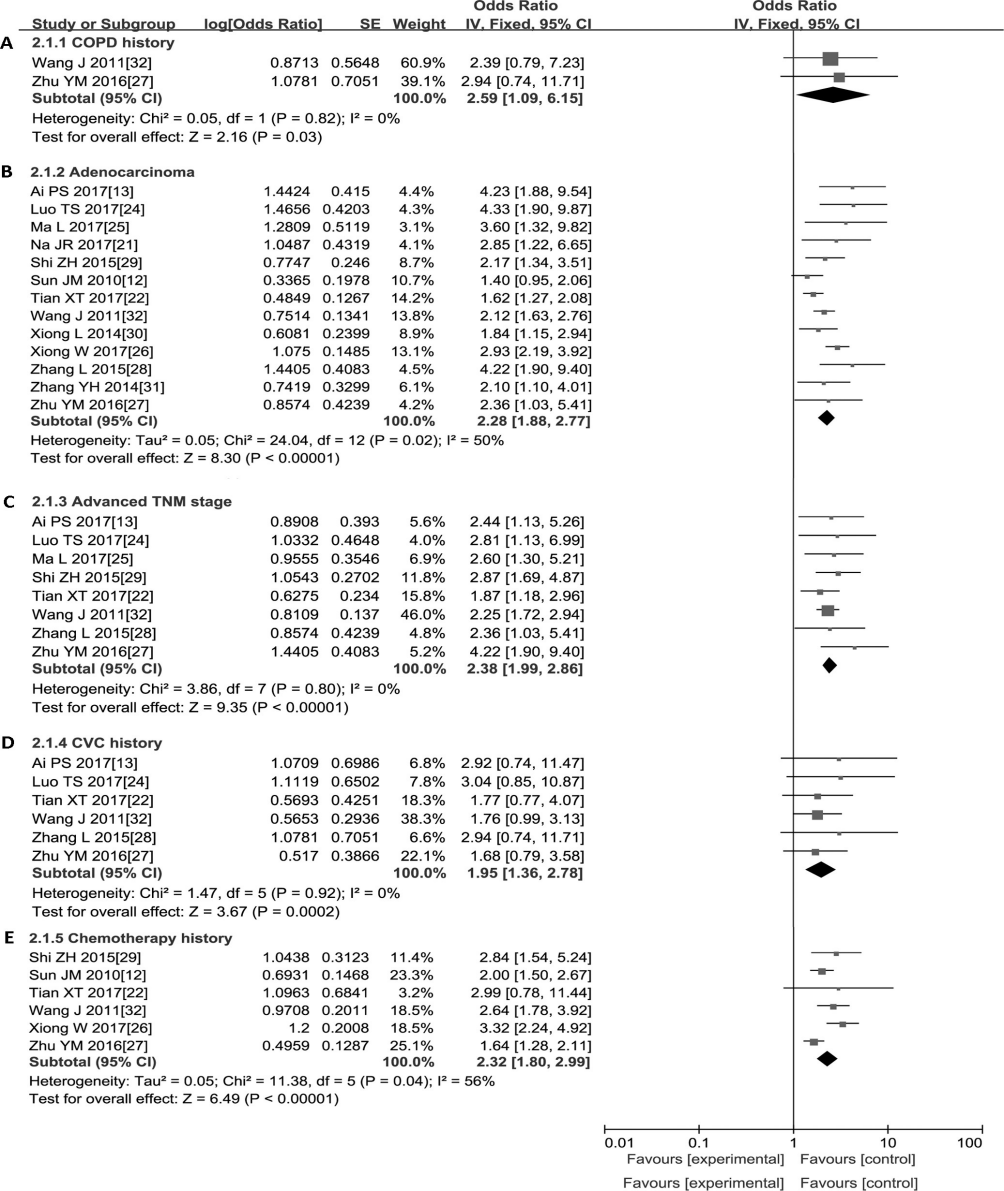
Forest plots of the clinical characteristics. (A) COPD history. (B) Adenocarcinoma. (C) Advanced TNM stage (III-IV). (D) CVC history. (E) Chemotherapy history. **Abbreviations:** COPD, chronic obstructive pulmonary disease; CVC, central venous catheter.

As shown in Fig 2, the meta-analysis of the relevant studies suggested that the prevalence of PE was significantly higher among LC patients with COPD history (OR = 2.59, 95% CI: 1.09, 6.15; P = 0.03), adenocarcinoma (OR = 2.28, 95% CI: 1.88, 2.77; P < 0.01), advanced TNM stage (III-IV vs. I-II, OR = 2.38, 95% CI: 1.99, 2.86; P < 0.01), CVC history (OR = 1.95, 95% CI: 1.36, 2.78; P < 0.01) and chemotherapy history (OR = 2.32, 95% CI: 1.80, 2.99, P < 0.01). In addition, a fixed-effects model was used because there was no obvious sample heterogeneity among the above mentioned studies, except for adenocarcinoma and chemotherapy history. To make our results comparable, a subgroup analysis comparing studies with similar adjusted variable data and dissimilar adjusted variable data was performed. As summarized in Table 2, the results of the two groups were consistent. Moreover, considering the difference in quality among the included studies, we conducted a subgroup analysis according to NOS quality scores, and the results based on low-quality studies were consistent with the results based on high-quality studies (Table 3).

**Table 2:** Clinical characteristic results stratified by the similarity of the adjusted variables of the studies included in the meta-analysis.

Study factors	Adjusted variables	No. of studies	OR (95% CI)	P	Heterogeneity		Model used
					I^2^ (%)	P_h_	
COPD history	Similar	2	2.59(1.09–6.15)	0.03	0%	0.82	Fixed
Adenocarcinoma	Similar	7	2.40(1.82–3.16)	<0.01	54%	0.04	Random
	Dissimilar	6	2.19(1.60–3.00)	<0.01	54%	0.06	Random
Advanced TNM stage	Similar	7	2.37(1.96–2.86)	<0.01	0%	0.70	Fixed
	Dissimilar	1	2.60(1.30–5.21)	<0.01	-	-	Fixed
CVC history	Similar	6	1.95(1.36–2.78)	<0.01	0%	0.92	Fixed
Chemotherapy history	Similar	4	1.99(1.63–2.42)	<0.01	49%	0.12	Fixed
	Dissimilar	2	2.53(1.54–4.15)	<0.01	76%	0.04	Random

**Abbreviations:** OR, odds ratio; 95% CI, 95% confidence interval; COPD, chronic obstructive pulmonary disease; CVC, central venous catheter.

**Table 3:** Clinical feature results stratified by the quality of the studies included in the meta-analysis.

Study factors	Study type	No. of studies	OR (95% CI) or	P	Heterogeneity		Model used
			WMD (95% CI)		I^2^ (%)	P_h_	
COPD history	Low-quality	1	2.94(0.74–11.71)	0.13	-	-	Fixed
	High-quality	1	2.39(0.79–7.23)	0.12	-	-	Fixed
Adenocarcinoma	Low-quality	5	2.58(1.89–3.51)	<0.01	20%	0.29	Fixed
	High-quality	8	2.15(1.70–2.71)	<0.01	60%	0.02	Random
Advanced TNM stage	Low-quality	3	2.90(1.83–4.59)	<0.01	0%	0.53	Fixed
	High-quality	5	2.30(1.89–2.80)	<0.01	0%	0.78	Fixed
CVC history	Low-quality	3	2.07(1.14–3.76)	0.02	0%	0.68	Fixed
	High-quality	3	1.88(1.21–2.93)	<0.01	0%	0.73	Fixed
Chemotherapy	Low-quality	1	1.64(1.28–2.11)	<0.01	-	-	Fixed
history	High-quality	5	2.49(2.07–3.01)	<0.01	13%	0.33	Fixed
WBC level	Low-quality	1	2.46(0.72–4.20)	<0.01	-	-	Fixed
	High-quality	4	0.33(-0.75–1.42)	0.55	69%	0.02	Random
Hb level	Low-quality	2	3.11(-50.13–56.34)	0.91	99%	<0.01	Random
	High-quality	4	-0.22(-0.50–0.05)	0.11	51%	0.10	Random
DD level	Low-quality	2	1.78(0.38–3.18)	0.01	94%	<0.01	Random
	High-quality	4	5.72(2.84–8.61)	<0.01	99%	<0.01	Random
PLT level	Low-quality	1	6.53(-29.27–42.33)	0.72	-	-	Fixed
	High-quality	4	-4.00(-21.87–13.87)	0.66	47%	0.13	Fixed
PaO_2_ level	Low-quality	1	-24.00(-33.11–14.89)	<0.01	-	-	Fixed
	High-quality	1	-27.00(-33.60–20.40)	<0.01	-	-	Fixed
CEA level	Low-quality	1	3.68(-10.94–18.30)	0.62	-	-	Fixed
	High-quality	1	10.30(9.95–10.65)	<0.01	-	-	Fixed

**Abbreviations:** OR, odds ratio; WMD, weighted mean difference; 95% CI, 95% confidence interval; COPD, chronic obstructive pulmonary disease; CVC, central venous catheter; WBC, white blood cell; Hb, haemoglobin; DD, D-dimer; PLT, platelet; PaO_2,_ partial pressure of oxygen; CEA, carcinoembryonic antigen.

### Meta-analysis of the clinical laboratory parameters

**Fig 3:**
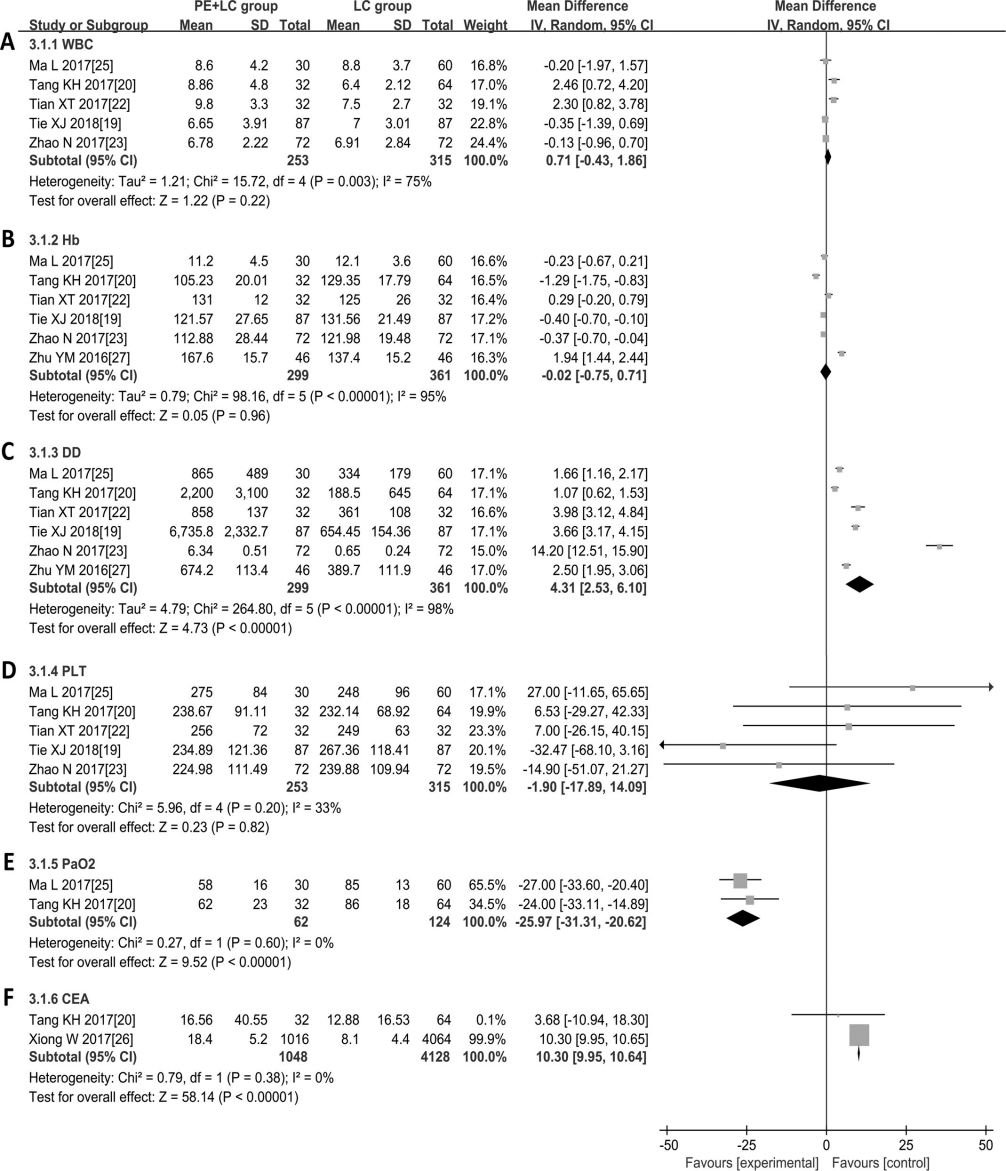
Forest plots of the clinical laboratory parameters. (A) WBC. (B) Hb. (C) DD. (D) PLT. (E) PaO_2_. (F) CEA. **Abbreviations:** PE + LC: pulmonary embolism with lung cancer patients; LC: sample lung cancer patients; WBC, white blood cell; Hb, haemoglobin; DD, D-dimer; PLT, platelet; PaO_2,_ partial pressure of oxygen; CEA, carcinoembryonic antigen.

As shown in Fig 3, the results showed that LC patients with PE had high levels of DD (WMD = 4.31, 95% CI: 2.53, 6.10; P < 0.01) and CEA (WMD = 10.30, 95% CI: 9.95, 10.64; P < 0.01) and a low level of PaO_2_ (WMD = -25.97, 95% CI: -31.31, -20.62; P < 0.01). However, differences in WBC (WMD = 0.71, 95% CI: -0.43, 1.86; P = 0.22), Hb (WMD = -0.02, 95% CI: -0.75, 0.71; P = 0.96) and PLT (WMD = -1.90, 95% CI: -17.89, 14.09; P = 0.82) levels between LC patients with and without PE were not significant. Furthermore, a random-effects model was utilized to analyse WBC, Hb and DD levels because there was obvious sample heterogeneity among the studies. In addition, the results based on the low-quality studies were consistent with the results based on the high-quality studies, except for WBC and CEA levels (Table 3).

### Meta-analysis of the survival rate at one year

**Fig 4:**
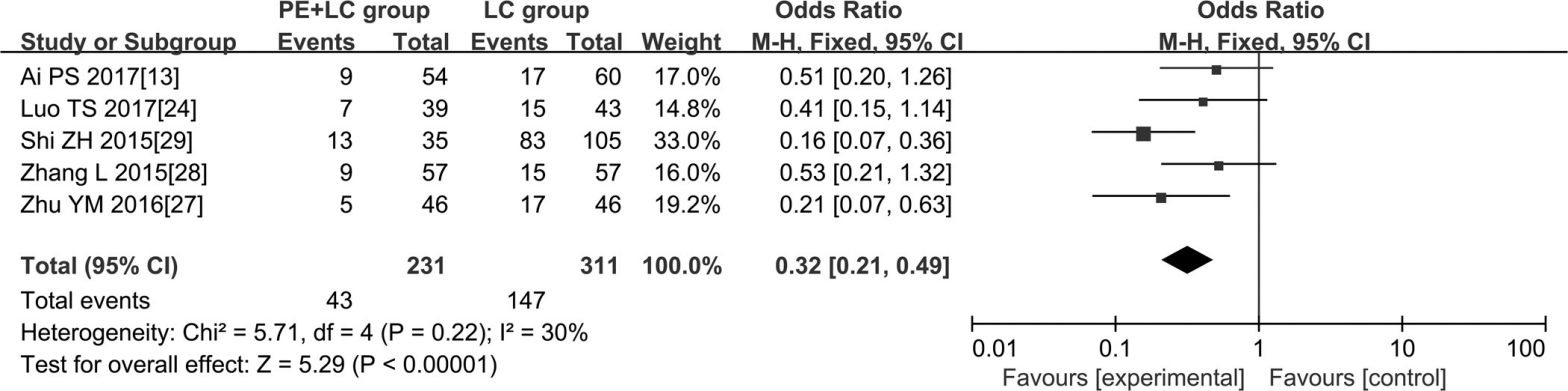
Forest plots of the survival rate at one year. **Abbreviations:** PE + LC: pulmonary embolism with lung cancer patients; LC: sample lung cancer patients.

Of the 16 included studies, only 5 investigated the survival rate of the patients at one year. Therefore, we used data from these studies to obtain the pooled survival rate at one year. As shown in Fig 4, the survival rate of patients with both PE and LC was significantly lower than that of patients with LC alone (OR = 0.32, 95% CI: 0.21, 0.49; P < 0.01), and heterogeneity among these studies was not significant (I^2^ = 30%, P = 0.22).

### Sensitivity analysis and publication bias

A sensitivity analysis was conducted to evaluate the influence of each included study, and the results showed that the heterogeneity, pooled ORs and WMDs of the indexes included above remained stable and were not significantly altered by any single study.

Begg’s test and Egger’s test were employed to assess publication bias in this meta-analysis. The results showed significant publication bias for CVC history, DD and PLT (P < 0.05).

## Discussion

We conducted this meta-analysis to clarify the clinical characteristics of LC patients with PE. The results showed that a high proportion of LC patients with PE had COPD history, adenocarcinoma, advanced TNM stage (III-IV), CVC history, chemotherapy history, high levels of DD and CEA and a low level of PaO_2_. Our findings may be significant for the identification of PE in LC patients at an early stage.

According to our study, the major histological type was adenocarcinoma in patients with LC and PE, which may be because adenocarcinoma cells can secrete mucin, potentially activating PLTs and other procoagulant factors and ultimately triggering PE [33–35]. Other studies have also reported that lung adenocarcinoma combined with PE is related to a hypercoagulable state [36] and EML4/ALK rearrangement [37] in these patients. COPD and advanced TNM stage have been reported to be associated with a hypercoagulable state and susceptibility to PE due to damage to the vascular endothelium and a high level of thrombogenesis, respectively [35,38,39]. Our study supports this viewpoint. Regarding treatment-related indicators, CVC and chemotherapy are increasingly being considered strong risk factors for PE in LC patients, which is also supported by our study, and may be due in part to the reduced production of endogenous anticoagulants and increased procoagulant activity [40–42]. While most research results confirm that surgery [13,19,21,27–30,32,43–46] and radiotherapy [22,27,29,32] history are not related to PE, other clinical features, such as obesity [22,27], anaemia (Hb < 100 g/L) [9,21,31,47], DVT [26], and hospitalization in the 12 months before the diagnosis of LC [48], may be associated with PE in LC patients. However, these indicators were not included in our meta-analysis due to the lack of statistical results derived from multivariate analyses. In addition, risk factors, including history of smoking, drinking, diabetes, hypertension, and cardiovascular diseases, have been explored [7,20–22,25,27,29,31,32,44,45,47,49,50], though these indicators were not found to be independent risk factors associated with PE in LC patients.

Regarding laboratory parameters, the results from low-quality and high-quality studies were consistent, except for the WBC levels not being associated with PE and CEA being associated with PE in the high-quality studies. Considering that high-quality studies had larger samples and their results are more likely to be reliable, we believe that a higher CEA is associated with a high risk of PE in LC patients but that WBC count is not. However, meta-analysis data on the association of most laboratory parameters and PE do not allow for definitive conclusions, as they suffer from publication bias (DD, PLT) or low study quality and small number of studies (PaO_2_, CEA, WBC). Therefore, more data are needed from high-quality studies to investigate the relationship between these laboratory parameters and PE considering that the number of articles available is currently small.

In addition, we confirmed previous reports that LC patients with PE have a significantly lower survival rate at one year than LC patients without PE. Although it seems inevitable, several factors could promote death in LC patients, but they were not distinguished in the studies included. Therefore, more clinical studies analysing PE-specific survival data are needed to directly clarify the prognostic value of PE in LC patients.

Our study inevitably had some shortcomings and omissions. First, the studies included in our meta-analysis were nearly all retrospective case-control studies, and the quality assessment by the NOS showed that the 16 included studies had relatively low scores (5~7). Second, confounding factors, such as the TNM stage, therapeutic schedule or other biomarkers, might also promote the occurrence of PE and affect the prognosis of LC patients; such an effect cannot be explored via subgroup analyses because the studies included did not provide sufficient information. Third, although the subgroup analysis results were consistent, the pooled multivariable estimated ORs may still suffer from confounding effects. In addition, all the included studies were published in English or Chinese, with most studies conducted in China, indicating that the results may have been subject to selection bias. Thus, more studies conducted in other countries are warranted to verify the association between the clinical features and PE risk in LC patients.

## Conclusions

In summary, our meta-analysis revealed that LC patients with PE have specific clinical features, including history of COPD, adenocarcinoma, more advanced TNM stage (III-IV), CVC, and chemotherapy history. Additionally, high levels of DD and CEA and a low level of PaO_2_ may be associated with PE in LC patients. However, a possible association between these clinical features and PE should be confirmed by more data of higher quality. Moreover, the presence of PE might significantly decrease the survival rate at one year among LC patients.

## Supporting information

S1 Table PRISMA 2009 checklist used in this meta-analysis. (DOC)

S2 Table Search terms and the number of studies identified from the PubMed database. (DOC)
