# Supplementary material for: Clinical features of pulmonary embolism in patients with lung cancer: A meta-analysis
Source: PLoS One. 2019 Sep 30;14(9):e0223230. doi: 10.1371/journal.pone.0223230 (PMC6768482; doi:10.1371/journal.pone.0223230)
Supplement: S2 Table — (DOC) [file pone.0223230.s002.doc]

**S2 Table. Search terms and the number of studies identified from the PubMed Database.**

| **Step** | **Search terms** | **Results** |
| --- | --- | --- |
| #1 | "Lung cancer"[MeSH Terms] | 219047 |
| #2 | (((Lung cancer[Title/Abstract] OR Lung carcinoma[Title/Abstract]) OR Lung tumor[Title/Abstract]) OR Lung neoplasm[Title/Abstract]) OR Lung oncology[Title/Abstract] | 154051 |
| #3 | (((Pulmonary cancer[Title/Abstract] OR Pulmonary carcinoma[Title/Abstract]) OR Pulmonary tumor[Title/Abstract]) OR Pulmonary neoplasm[Title/Abstract]) OR Pulmonary oncology[Title/Abstract] | 3404 |
| #4 | #1 OR #2 OR #3 | 268445 |
| #5 | "Pulmonary embolism"[MeSH Terms] | 37349 |
| #6 | ((Pulmonary embolism[Title/Abstract] OR Pulmonary thromboembolism[Title/Abstract]) OR Pulmonary thrombosis[Title/Abstract]) OR PE[Title/Abstract] | 62971 |
| #7 | (Lung embolism[Title/Abstract] OR Lung thromboembolism[Title/Abstract]) OR Lung thrombosis[Title/Abstract] | 342 |
| #8 | #5 OR #6 OR #7 | 78781 |
| #9 | #4 AND #8 AND ("00001/01/0"[Date - Publication] : "2019/02/01"[Date - Publication]) | 904 |

**Final search strategy:**

((("Lung cancer"[MeSH Terms] OR ((((Lung cancer[Title/Abstract] OR Lung tumor[Title/Abstract]) OR Lung carcinoma[Title/Abstract]) OR Lung neoplasm[Title/Abstract]) OR Lung oncology[Title/Abstract])) OR ((((Pulmonary cancer[Title/Abstract] OR Pulmonary tumor[Title/Abstract]) OR Pulmonary carcinoma[Title/Abstract]) OR Pulmonary neoplasm[Title/Abstract]) OR Pulmonary oncology[Title/Abstract])) AND (("Pulmonary embolism"[Mesh] OR (((Pulmonary embolism[Title/Abstract] OR Pulmonary thromboembolism[Title/Abstract]) OR Pulmonary thrombosis[Title/Abstract]) OR PE[Title/Abstract])) OR ((Lung embolism[Title/Abstract] OR Lung thromboembolism[Title/Abstract]) OR Lung thrombosis[Title/Abstract]))) AND ("00001/01/0"[Date - Publication] : "2019/02/01"[Date - Publication])
